# Stereotactic Body Radiotherapy for Frail Patients with Primary Renal Cell Carcinoma: Preliminary Results after 4 Years of Experience

**DOI:** 10.3390/cancers13133129

**Published:** 2021-06-23

**Authors:** Laure Grelier, Michael Baboudjian, Bastien Gondran-Tellier, Anne-Laure Couderc, Robin McManus, Jean-Laurent Deville, Ana Carballeira, Raphaelle Delonca, Veronique Delaporte, Laetitia Padovani, Romain Boissier, Eric Lechevallier, Xavier Muracciole

**Affiliations:** 1 Department of Urology and Kidney Transplantation, Aix-Marseille University, Assistance Publique–Hôpitaux de Marseille (AP-HM), Conception Academic Hospital, 13005 Marseille, France; Laure.GRELIER@ap-hm.fr (L.G.); Laetitia.Padovani@ap-hm.fr (L.P.); Xavier.MURACCIOLE@ap-hm.fr (X.M.); 2 Department of Radiotherapy, La Timone Hospital, Assistance Publique-Hopitaux de Marseille (AP-HM), 13005 Marseille, France; bastien.gondran@ap-hm.fr (B.G.-T.); robinmcmanus@rcsi.ie (R.M.); raphaelle.delonca@ap-hm.fr (R.D.); Veronique.DELAPORTE@ap-hm.fr (V.D.); romain.boissier@ap-hm.fr (R.B.); eric.lechevallier@ap-hm.fr (E.L.); 3 Internal Medicine, Geriatrics and Therapeutic University, Assistance Publique–Hopitaux de Marseille (AP-HM), 13005 Marseille, France; Anne-Laure.COUDERC@ap-hm.fr; 4 Department of Oncology, La Timone Hospital, Aix-Marseille University, Assistance Publique–Hopitaux de Marseille (AP-HM), 13005 Marseille, France; Jean-laurent.DEVILLE@ap-hm.fr; 5 Department of Radiology, Aix-Marseille University, Assistance Publique–Hopitaux de Marseille (AP-HM), Conception Academic Hospital, 13005 Marseille, France; ana.carballeira-alvarez@ap-hm.fr

**Keywords:** stereotactic body radiotherapy, renal cell carcinoma, frail patients, oncological outcomes

## Abstract

**Simple Summary:**

Surgical therapy is currently the standard of care for the treatment of primary renal cell carcinoma (RCC). Alternative strategies such as stereotactic body radiotherapy (SBRT) have emerged as potentially curative treatment approaches. In this study, we show a promising short-term local control effect of SBRT in the management of primary RCC. The treatment was well tolerated with no high-grade side effects. The main advantages are the outpatient management without anesthesia and the non-invasive approach. Thus, SBRT appears to be a promising alternative to surgery, or ablative therapy, to treat primary RCC in patients with poor physical health. Future studies are needed to definitively assess the place of SBRT in the RCC treatment portfolio.

**Abstract:**

Introduction: The aim of this study was to report the oncological outcomes and toxicity of stereotactic body radiotherapy (SBRT) to treat primary renal cell carcinoma (RCC) in frail patients unfit for surgery or standard alternative ablative therapies. Methods: We retrospectively enrolled 23 patients who had SBRT for primary, biopsy-proven RCC at our tertiary center between October 2016 and March 2020. Treatment-related toxicities were defined using CTCAE, version 4.0. The primary outcome was local control which was defined using the Response Evaluation Criteria in Solid Tumors. Results: The median age, Charlson score and tumor size were 81 (IQR 79–85) years, 7 (IQR 5–8) and 40 (IQR 28–48) mm, respectively. The most used dose fractionation schedule was 35 Gy (78.3%) in five or seven fractions. The median duration of follow-up for all living patients was 22 (IQR 10–39) months. Local recurrence-free survival, event-free survival, cancer-specific survival and overall survival were 96 (22/23), 74 (18/23), 96 (22/23) and 83% (19/23), respectively. There were no grade 3–4 side effects. No patients required dialysis during the study period. No treatment-related deaths or late complications were reported. Conclusion: SBRT appears to be a promising alternative to surgery or ablative therapy to treat primary RCC in frail patients.

## 1. Introduction

Renal cell carcinoma (RCC) represents around 3% of all cancers, with the highest incidence occurring in Western countries [1]. Surgical therapy is currently the standard of care for the treatment of primary RCC in fit patients with adequate renal function [2]. Given the demographics of patients with RCC, many older patients have comorbidities which may preclude them from major surgery. Alternative strategies such as cryotherapy and radiofrequency ablation have emerged as potentially curative treatment approaches for patients who refuse or are unsuitable for surgery [3,4,5]. However, these minimally invasive therapies are limited to small renal masses, distant from vascular structures and the upper urinary tract [6,7]. By contrast, stereotactic body radiotherapy (SBRT) is an emerging noninvasive treatment and does not necessitate inpatient hospital treatment. SBRT is delivered in single or multiple treatment sessions, and is typically associated with low toxicity and excellent local control rates in a variety of malignancies [8].

RCC is usually considered resistant to radiation delivered using conventional fractionation schedules (1.8–2 Gy per fraction). The current literature reports encouraging results of SBRT on primary RCC in terms of local control and acceptable toxicity [9]. However, there is still insufficient evidence to recommend this therapy.

The aim of our study was to report the oncological outcomes and toxicity of SBRT for frail patients with primary RCC unfit for surgery or standard alternative ablative therapies.

## 2. Materials and Methods

### 2.1. Population Study

The study was approved by the Ethics Committee of the French Urological Association (CERU_2020/014). We retrospectively reviewed the charts of all patients who had SBRT for primary RCC at our tertiary center between October 2016 and March 2020. Data were extracted from medical files and collected in a pseudo-anonymized database in accordance with GDPR regulations. Eligible patients were included who were medically unfit for surgery and were poor candidates for cryotherapy and radiofrequency ablation (tumor size > 4 cm, near from vascular pedicle or upper urinary tract). Patients who could not tolerate the prolonged supine positioning necessary for successful treatment, or with a history of abdominal or pelvic radiotherapy were excluded. No restrictions were applied regarding the histological subtype, size or stage of the tumor. All patients had a proven histological diagnosis of RCC by percutaneous tumor biopsy under computed tomography (CT) guidance and all indications for radiotherapy were confirmed by our multidisciplinary team (MDT).

### 2.2. Stereotactic Body Radiotherapy

Patients were positioned with a TomoTherapy™ (Accuray, Madison, WI, USA) device, which delivered arc x-ray therapy of 6 MV. Prior to CT, patients were immobilized with the BlueBAG™ (Elekta, Stockholm, Sweden) BodyFIX Vacuum Cushions system and the abdominal compression plate (ACP) to minimize breathing motion. The gross tumor volume (GTV) was contoured on different reconstructions of the simulation CT with breath-phased 3D CT scan to encompass the motion and to create an internal target volume (ITV). For the last patients included, the GTV was contoured on the different respiratory phases of a 4D simulation scan. A 5 mm expansion was given to derive the planning target volume (PTV). The dosimetry was evaluated on Accuray’s VOLO™ (Accuray, Madison, WI, USA) software. The objective prescription isodose was 90%. Sessions were spaced 48 h apart and were delivered on non-consecutive days (one day interval). SBRT procedures were adapted from consensus statements from the International Radiosurgery Oncology Consortium for primary renal cell carcinoma [10,11]. The total dose administered was in accordance with De Meerleer’s guidelines [12].

### 2.3. Follow-up and Endpoints

Patients were followed up with every 3 months for the first two years, and twice annually thereafter. The follow-up included physical examination, glomerular filtration rate (eGFR) which was estimated by the Chronic Kidney Disease Epidemiology Collaboration (CKD-EPI) equation, and radiological examination with CT-scan, MRI or ultrasound. Treatment-related toxicities were defined using Common Terminology Criteria for Adverse Events, version 4.0. The primary outcome was local control, which was defined using Response Evaluation Criteria in Solid Tumors, version 1.1 [13]. Secondary outcomes included treatment-related toxicities, evolution of renal function, event-free survival, cancer-specific survival and overall survival.

### 2.4. Statistical Analysis

Descriptive statistics were delineated for the available variables. Quantitative variables were reported as median and interquartile range (IQR) and analyzed by Mann–Whitney U Test. Categorical variables were described as numbers and percentages and were analyzed by Chi-squared test. Kaplan–Meier curves were generated for all time-to-event endpoints. Statistical analyses were performed using R Version 4.0.2 (Foundation for Statistical Computing, Vienna, Austria). A *p*-value of ≤ 0.05 was considered statistically significant.

## 3. Results

Between October 2016 and March 2020, 24 patients underwent SBRT for primary RCC in our center and were assessed for eligibility. One patient declined to continue radiation therapy after initial sessions and was excluded. Twenty-three patients were included in the final analysis.

### 3.1. Baseline Characteristics

Patient and tumor characteristics are summarized in Table 1. The median age in the study cohort was 81 (IQR 79–85) years. Eight female patients (34.8%) were included and the median Charlson Comorbidity score was 7 (IQR 5–8). Pathologic confirmation before treatment was achieved in all cases. Overall, 73.9% (17/23) of patients harbored clear cell RCC. Stages T1a, T1b and T2 were recorded in 56.5, 39.1 and 4.4% of cases, respectively. A high Fuhrman grade (G3–4) was recorded in 30.4% of all patients. Four patients (17.4%) had M1 disease. The median maximal tumor size was 40 (IQR 28–48) mm.

Table 2 shows dose fractionation schedules. The most used dose schedule was 35 Gy (78.3%) in five fractions (43.5%, 10/23) or seven fractions (34.8%, 8/23). No complications or technical difficulties were recorded during sessions.

### 3.2. Recurrence and Survival

**Figure 1:**
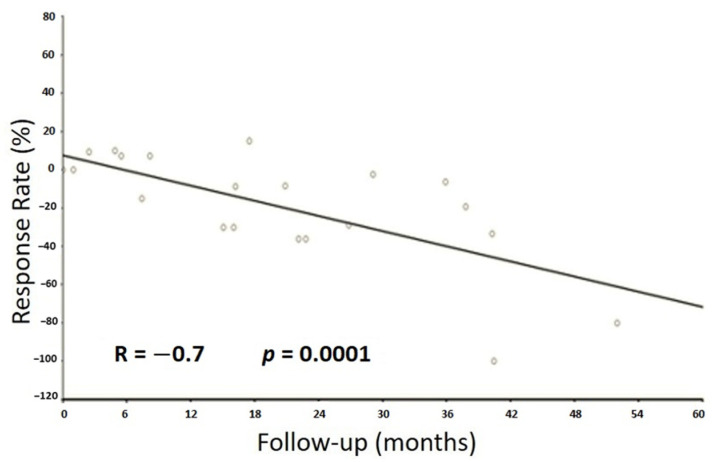
Correlation between tumor size and follow-up.

The median duration of follow-up for all living patients was 22 (IQR 10–39) months. At the time of most recent follow-up, we did not reach 50% recurrence, events or death in the study group. One patient experienced local recurrence after 36 months of follow-up. We observed a significant correlation between time of follow-up and decreased size of primary tumor (Figure 1).

**Figure 2:**
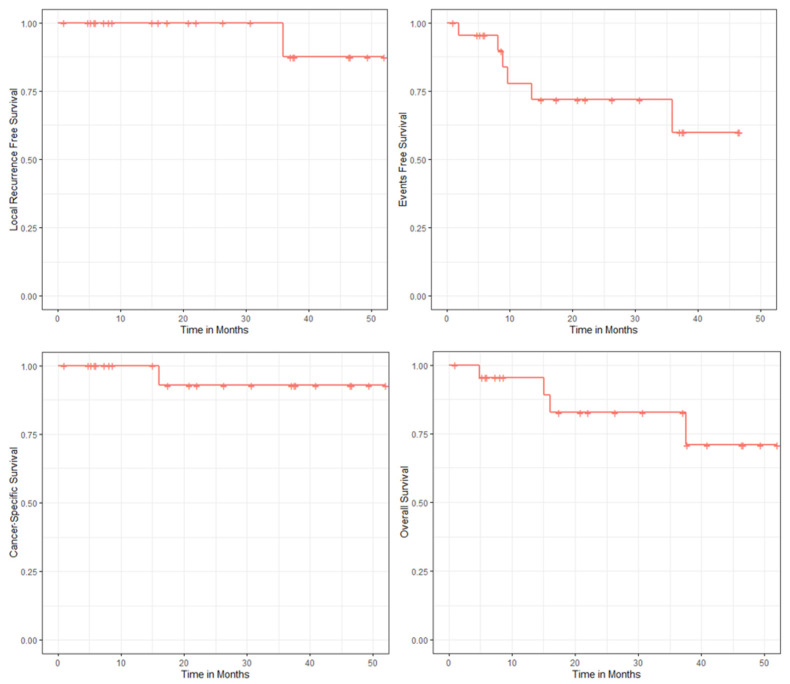
Kaplan–Meier estimates of local recurrence-free survival; event-free survival; cancer-specific survival and overall survival.

Lymph node and metastatic recurrences were recorded in one and four cases, respectively, including two cases in patients who were already M1 before SBRT. A total of four deaths were observed: one case related to disease progression and three deaths from another cause. Thus, after a median follow-up of 22 months, local recurrence-free survival, event-free survival, cancer-specific survival and overall survival were 96, 74, 96 and 83%, respectively (Figure 2).

### 3.3. Evolution of Renal Function

The median baseline eGFR was 57 (IQR 34–75) mL per minute. The median change in eGFR at the most recent follow-up was −7 (IQR −17; 0) mL per minute (*p* = 0.15). No patients required dialysis during the study period.

### 3.4. Toxicity

A total of five side effects (21.7%) were recorded. Grade 2 asthenia was observed in two cases. One grade 2 epigastralgia and two grade 1 episodes of nausea were also recorded. There were no grade 3–4 side effects recorded. No treatment-related deaths or late complications were recorded.

## 4. Discussion

Over the past decade, SBRT has emerged as a potential treatment option for primary RCC [4]. Defining a potential target population for SBRT is a major challenge. The main advantages are the outpatient management without anesthesia and the non-invasive approach. The majority of patients were either old patients with multiple comorbidities, or patients with tumors inaccessible to standard focal therapies. SBRT has been implemented in our practices from 2016 to treat primary RCC in patients with poor physical health who were medically inoperable and unfit for cryotherapy and radiofrequency ablation. To our knowledge and following a review of the literature, we report the first series of SBRT in this patient population.

Our study reports the promising short-term local control effects of SBRT with a TomoTherapy™ (Accuray, Madison, WI, USA) device. After a median follow-up of 22 months, only one patient (4%) experienced local recurrence. In this case, a percutaneous biopsy of the tumor was performed because intratumoral hypermetabolic foci increased and was positive without an increase in tumor size. Our results correlate with previous data from Chang et al. who reported no local recurrence after a median follow-up of 19 months in their series of 16 patients treated with 30 to 40 Gy in five fractions [14]. In a prospective cohort of 37 patients receiving either a single fraction of 26 Gy or three fractions of 14 Gy for inoperable renal cell carcinoma, Siva et al. reported a freedom from local progression at 2 years of 100% [15]. Additionally, our results support those of a meta-analysis which enrolled 126 patients from 10 studies [9]. The most commonly employed fractionation schedule was 40 Gy, delivered over five fractions, and a local control was reported of 93.91% (range: 84–100%) [9]. A pooled multi-institutional analysis of IROCK focused on a cohort of 223 patients treated for primary RCC by stereotaxic ablative radiotherapy [11]. The patients were divided into two groups: those receiving a single fraction with an average dose of 25 Gy (14–26 Gy), and those receiving multiple fractions with an average total dose of 40 Gy (2 to 10 fractions). The local control rate at five years was 97.8% and was similar between both groups. The authors reported in the group of patients treated with a multifractional regimen a significantly lower rate of nausea but a poorer progression-free survival and cancer-specific survival. The authors raised the possibility of an abscopal effect in the monofraction group. The immunosensitive nature of CCR was also mentioned in the study by Siva et al. [15]. The possibility of SBRT-mediated immunomodulation is based on preclinical in vivo data and then on reported cases of patients with an abscopal effect [16,17]. Our team modeled the synergy between SBRT and checkpoint inhibitor therapies, emphasizing the advantage of delivering these two treatments concomitantly [18]. In addition, we developed a new concept of immunologically effective dose (IED) varying with the radiotherapy regimen used, complementary to the classic linear–quadratic formula [19]. In our series, some patients presented with an increase in tumor size 12–15 months after SBRT sessions no greater than 20%, but decrease after that time, as reported by Ponski et al. [20] and Chang J.H. [14]. This initial, and temporary, increase in tumor size could be explained by an immunological stimulating effect of SBRT.

The safety of SBRT was confirmed in the present study; 21.7% grade 1–2 side effects with no grade 3 or higher event occurring. Our results are very similar to previous data from Siva et al. [15] and Correa et al. [21], which reported 3 and 1.5% occurrence, respectively, of grade ≥3 side effects. Furthermore, SBRT had a modest impact on renal function, with a mean reduction in eGFR at last follow-up of −7 mL/min. These outcomes are in agreement with previous data [22] and comparable to those of nephron-sparing approaches such as PN or ablative therapies [23,24,25].

The present study has several limitations that should be acknowledged. The main limitations concern its retrospective design, the small cohort included and the short follow-up. Conversely, the main strength of this preliminary study was to report the outcomes of SBRT in a cohort of frail patients which has not previously been reported in the literature. All patients presented with a progressive tumor after active surveillance and/or a tumor with high histological grade. Future studies are essential to determine the place of SBRT in the therapeutic arsenal of RCC. An ongoing clinical trial will provide additional important findings on the subject [26]. To date, there is no prospective randomized study comparing SBRT to standard ablative therapies. The meta-analysis by Kunkle et al. [5] that included 1375 primary RCC patients treated with radiofrequency ablation or cryoablation reported a local control rate of 87.1 and 94.8%, respectively, with a median follow-up of 18.7 months. In addition, the average tumor diameter was 26 mm, against 40mm in our study.

## 5. Conclusions

SBRT appears to be a promising alternative to surgery or ablative therapy to treat primary RCC in patients with poor physical health. Future studies are needed to definitively assess the place of SBRT in the RCC treatment portfolio.

## Figures and Tables

**Table 1:** Baseline characteristics.

Variables	Overall Cohort (*n* = 23)
Gender, *n (%)*	
Male	15 (65.2)
Female	8 (34.8)
Median (IQR) age, years	81 (79–85)
ECOG performance score, *n (%)*	
0	10 (43.5)
1	11 (47.8)
≥2	2 (8.7)
Median (IQR) Charlson score	7 (5–8)
Comorbidities, *n (%)*	
Diabetes mellitus	8 (34.8)
Arterial hypertension	14 (60.9)
Median (IQR) creatinine clearance, mL/mn/1.73 m²	57 (34–75)
Tumor stage, *n (%)*	
T1a	13 (56.5)
T1b	9 (39.1)
T2	1 (4.4)
Metastatic stage, *n (%)*	
M0	19 (82.6)
M1	4 (17.4)
Tumor side, *n (%)*	
Right	13 (56.5)
Left	10 (43.5)
Fuhrman grade, *n (%)*	
Low grade (1–2)	14 (60.9)
High grade (3–4)	7 (30.4)
Unknown	2 (8.7)
RCC type, *n (%)*	
Clear cell	17 (73.9)
Papillary	2 (8.7)
Other	4 (17.4)
Median (IQR) tumor size, mm	40 (28–48)

Legend: ECOG: Eastern Cooperative Oncology Group; RCC: renal cell carcinoma.

**Table 2:** Dose fractionation schedules.

Dose (Gy)/Fraction (Fr)	*n* (%)
35 Gy/5 Fr	10 (43.5)
35 Gy/7 Fr	8 (34.8)
36 Gy/3 Fr	4 (17.4)
24 Gy/3 Fr	1 (4.3)

Legend: Gy: gray; Fr: fraction.

## Data Availability

The data presented in this study are available on request from the corresponding author.
